# Explainable coronary artery disease prediction model based on AutoGluon from AutoML framework

**DOI:** 10.3389/fcvm.2024.1360548

**Published:** 2024-07-01

**Authors:** Jianghong Wang, Qiang Xue, Chris W. J. Zhang, Kelvin Kian Loong Wong, Zhihua Liu

**Affiliations:** ^1^ Faculty of Information Engineering and Automation, Center for Precision Medicine, Yan'an Hospital of Kunming City & Kunming University of Science and Technology, Kunming, China; ^2^ Department of Mechanical Engineering, University of Saskatchewan, Saskatoon, SK, Canada; ^3^ Bayer HealthCare & Dana-Farber Cancer Institute, Harvard University, Boston, MA, United States

**Keywords:** Automated Machine Learning, AutoGluon, SHapley Additive exPlanations, heart disease, prediction model

## Abstract

**Objective:**

This study focuses on the innovative application of Automated Machine Learning (AutoML) technology in cardiovascular medicine to construct an explainable Coronary Artery Disease (CAD) prediction model to support the clinical diagnosis of CAD.

**Methods:**

This study utilizes a combined data set of five public data sets related to CAD. An ensemble model is constructed using the AutoML open-source framework AutoGluon to evaluate the feasibility of AutoML in constructing a disease prediction model in cardiovascular medicine. The performance of the ensemble model is compared against individual baseline models. Finally, the disease prediction ensemble model is explained using SHapley Additive exPlanations (SHAP).

**Results:**

The experimental results show that the AutoGluon-based ensemble model performs better than the individual baseline models in predicting CAD. It achieved an accuracy of 0.9167 and an AUC of 0.9562 in 4-fold cross-bagging. SHAP measures the importance of each feature to the prediction of the model and explains the prediction results of the model.

**Conclusion:**

This study demonstrates the feasibility and efficacy of AutoML technology in cardiovascular medicine and highlights its potential in disease prediction. AutoML reduces the barriers to model building and significantly improves prediction accuracy. Additionally, the integration of SHAP enhances model transparency and explainability, which is critical to ensuring model credibility and widespread adoption in cardiovascular medicine.

## 1 Introduction

In recent years, incidences and death tolls of cardiovascular disease (CVD) have been increasing, making them one of the leading causes of mortality and morbidity worldwide. The American Heart Association (AHA), in their 2023 CVD statistics report, highlighted that in the initial year of the COVID-19 pandemic in 2020, the U.S. witnessed a dramatic increase in CVD-related deaths (1). Fatalities surged from 876,613 in 2019 to 928,741 in 2020, surpassing 910,000 in 2003. The “Report on Cardiovascular Health and Diseases in China 2021: An Updated Summary” indicates that approximately 330 million people in China are affected by CVD. In 2019, CVD fatalities accounted for 46.74% and 44.26% of total deaths in rural and urban areas, respectively (2). One in every five deaths was attributed to CVD. Coronary Artery Disease (CAD) is a cardiovascular disease caused by the hardening and narrowing of the arteries in the coronary arteries, leading to insufficient blood supply to the heart. It is one of the most common and fatal diseases globally. Therefore, accurate screening of potential patients holds paramount theoretical and practical significance.

Machine learning is a technique of artificial intelligence characterized by algorithms that enable machines to learn and improve autonomously. Combining machine learning and medical data can yield unexpected results, assisting physicians in diagnostic decision-making (3, 4). Multiple studies have explored and implemented machine learning methods for predicting the risk of CAD. Within these studies, researchers have assessed different machine learning algorithms using specific data sets and identified models with optimal performance on test sets. K-nearest neighbor, random forest, logistic regression, and neural networks have obtained the highest classification performance in various studies (5–8). Several studies have concentrated on enhancing machine learning approaches by utilizing optimization strategies to improve prediction accuracy on test sets. For instance, machine learning algorithms that leverage particle swarm and ant colony optimization have been employed to predict and classify heart disease (9). By combining the multi-objective particle swarm optimization and random forest, a new approach is proposed to predict heart disease (10). The advanced particle swarm optimization merged lion algorithm is used to improve heart disease prediction, and the performance is better than other traditional models (11). Bayesian optimization approaches have been implemented to fine-tune the hyperparameter settings of XGBoost in producing heart disease prediction models (12). Furthermore, other analyses have examined multi-model ensemble techniques (13–16). Findings suggest that model integration can significantly enhance predictive performance, particularly in accurately identifying CAD, showing excellent results.

In the context of disease prediction, machine learning algorithms can analyze vast amounts of patient data, uncovering and extracting vital features related to disease, thus predicting the likelihood of a patient developing disease. However, employing machine learning methods necessitates manual model selection and hyperparameter tuning, requiring a profound understanding and expertise of the algorithms. Automated Machine Learning (AutoML) is a technique that automates machine learning model development using machine learning algorithms (17). It seeks to reduce the need for human intervention in the model development process, thereby accelerating model development and deployment (18). AutoML has extensive applications in diagnostics and forecasting in medicine. A model developed using AutoML to predict the survival rate of COVID-19 patients has demonstrated that AutoML is an efficient method for generating clinical decision support tools based on machine learning (19). An AutoML model based on the XGBoost algorithm, designed to predict the 30-day mortality rate of non-cholestatic cirrhosis patients, outperforms existing scoring systems (20). Furthermore, an AutoML model based on the GBM algorithm for the early identification of critically ill acute pancreatitis patients hospitalized due to acute pancreatitis has shown significant clinical utility (21). Additionally, AutoML-based models have exhibited excellent performance in predicting the recurrence of common bile duct stones after endoscopic retrograde cholangiopancreatography treatment (22). AutoML has also shown remarkable performance in predicting the 90-day mortality rate of gastric cancer patients who have undergone gastrectomy with a large sample size (23).

Artificial Intelligence has tremendous potential in the medical field, but its lack of transparency has limited its adoption in clinical practice. Explainable AI holds the potential to overcome this issue (24). In medical diagnostic research, the explainability of the model is crucial. Providing a clear, transparent, and logically coherent rationale is essential when a model generates predictions or recommendations. This ensures physicians can trust the model's outputs and make informed clinical decisions (25).

Machine learning offers multidimensional possibilities in medical data analysis and mining. With the continuous updating and advancement of machine learning technology, the requirement for practitioners' professional knowledge is also increasing. AutoML significantly reduces the threshold and burden of modelers through automatic modeling and parameter optimization. To investigate the disease prediction effect of AutoML technology in cardiovascular medicine, our study utilized publicly available clinical data on heart disease and used AutoGluon, an AutoML framework based on multi-model fusion technology, to construct prediction models. In addition, the explainability of the model was crucial for clinical prediction. Most of the CAD prediction models built in previous studies were not analyzed for explainability, making it difficult to explain the decision-making process of the models. To improve the explainability of our model, this study utilizes SHapley Additive exPlanations (SHAP) for analysis, which provides an in-depth understanding of the contribution of each input feature to the model. Our study aims to explore the potential of utilizing AutoML techniques to construct explainable CAD prediction models.

## 2 Materials and methods

### 2.1 Data preparation

This study utilizes a combined data set on heart disease (26). The data set amalgamates information from five distinct data sets: Cleveland (303 observations), Hungary (294 observations), Switzerland (123 observations), VA Long Beach (200 observations), and Statlog (270 observations). The original data can be accessed through the University of California, Irvine Machine Learning Repository (Cleveland, Hungary, Switzerland, VA Long Beach: https://archive.ics.uci.edu/dataset/45/heart+disease, Statlog: https://archive.ics.uci.edu/dataset/145/statlog+heart). The data are obtained from public repositories and do not contain identifiable private information. This data set is licensed under the Creative Commons Attribution 4.0 International License (CC BY 4.0). We confirm that this study adheres to the principles of the Helsinki Declaration. The combined data set comprises 11 features for predicting CAD, yielding 918 observations after removing duplicates. Among these are 508 instances of CAD and 410 instances without CAD. Categorical variables within the data set undergo ordinal encoding. The processed variables are shown in Table 1. In the data set, a CAD status value of 0 signifies non-CAD patients (arterial diameter narrowing less than 50%), while 1 indicates CAD patients (arterial diameter narrowing greater than 50%). This diagnostic outcome is derived from invasive angiographic examinations of coronary arteries. The original data set contains missing values for cholesterol. We address these missing values by using mean imputation. The baseline comparison table between patients with CAD and patients without CAD is shown in Table 2. It is worth noting that inherent limitations may exist due to using a data set sourced from an open-access website and its limited scope. These limitations include significant disparities and a lack of propensity matching, as well as the absence of BMI, diabetes, and other variables. Additionally, there is a differentiation between prevalent and incidental cases and a deficiency of appropriate data regarding potential confounding factors.

**Table 1:** Description of variables in the data set.

Variable	Variable description	Type
Age	Age [years]	Quantitative
Sex	Sex [1: male, 0: female ]	Qualitative
Chest pain type	Chest pain type [0: typical angina, 1: atypical angina, 2: Non-anginal pain, 3: Asymptomatic]	Qualitative
Resting BP	Resting blood pressure [mmHg]	Quantitative
Cholesterol	Serum cholesterol [mg/dl]	Quantitative
Fasting blood sugar	Fasting blood sugar [1: Fasting blood sugar > 120 mg/dl, 0: Fasting blood sugar ≤ 120 mg/dl]	Qualitative
Resting ECG	Resting electrocardiogram results [0: Normal, 1: Having ST-T wave abnormality (T wave inversions and/or ST elevation or depression of >0.05 mV), 2: Showing probable or definite left ventricular hypertrophy by Estes’ criteria]	Qualitative
Max HR	Maximum heart rate achieved [between 60 and 202, bpm]	Quantitative
Exercise angina	Exercise-induced angina [1: yes, 0: no]	Qualitative
Old peak	ST depression induced by exercise relative to rest [mm]	Quantitative
ST slope	The slope of the peak exercise ST segment [0: up-sloping, 1: Flat, 2: down-sloping]	Qualitative
CAD status	Output class [1: CAD, 0: non-CAD]	Qualitative

**Table 2:** Baseline comparison table between patients with CAD and non-patients.

Variable	Overall (*n* = 918)	CAD (*n* = 508)	Non-CAD (*n* = 410)	*p*-value
Qualitative data: *n* (%)				
Sex				
Male	725 (79.0%)	458 (90.2%)	267 (65.1%)	< 0.001
Female	193 (21.0%)	50 (9.8%)	143 (34.9%)	
Chest pain type				
ATA (Atypical angina)	173 (18.8%)	24 (4.7%)	149 (36.3%)	< 0.001
NAP (Non-anginal pain)	203 (22.1%)	72 (14.2%)	131 (32.0%)	
ASY (Asymptomatic)	496 (54.0%)	392 (77.2%)	104 (25.4%)	
TA (Typical angina)	46 (5.0%)	20 (3.9%)	26 (6.3%)	
Fasting blood sugar				
≤120 mg/dl	704 (76.7%)	338 (66.5%)	366 (89.3%)	< 0.001
>120 mg/dl	214 (23.3%)	170 (33.5%)	44 (10.7%)	
Resting ECG (resting electrocardiogram results)				
Normal	552 (60.1%)	285 (56.1%)	267 (65.1%)	< 0.001
ST (having ST-T wave abnormality)	178 (19.4%)	117 (23.0%)	61 (14.9%)	
LVH (left ventricular hypertrophy)	188 (20.5%)	106 (20.9%)	82 (20.0%)	
Exercise angina				
Yes	371 (40.4%)	316 (62.2%)	55 (13.4%)	< 0.001
No	547 (59.6%)	192 (37.8%)	355 (86.6%)	
ST slope (the slope of the peak exercise ST segment)				
Up-sloping	395 (43.0%)	78 (15.4%)	317 (77.3%)	< 0.001
Flat	460 (50.1%)	381 (75.0%)	79 (19.3%)	
Down-sloping	63 (6.9%)	49 (9.6%)	14 (3.4%)	
Quantitative data: mean (SD)				
Age (years)	53.51 (9.43)	55.90 (8.73)	50.55 (9.44)	< 0.001
Resting BP (resting blood pressure, mmHg)	132.40 (18.51)	134.19 (19.83)	130.18 (16.50)	< 0.001
Cholesterol (serum cholesterol, mg/dl)	245.57 (53.38)	251.06 (52.27)	238.77 (54.02)	< 0.001
Max HR (maximum heart rate achieved, bpm)	136.80 (25.46)	127.66 (23.39)	148.15 (23.29)	< 0.001
Old peak (ST depression induced by exercise relative to rest, mm)	0.89 (1.07)	1.27 (1.15)	0.41 (0.70)	< 0.001

### 2.2 AutoGluon: AutoML framework

AutoGluon is an AutoML framework developed by Amazon Web Services (AWS) (27). Its core idea is to simplify the selection, training, and deployment of machine learning models so that a wide range of developers can easily apply powerful machine learning techniques without needing to understand low-level details. First, AutoGluon offers automated feature generation, selection, and transformation, which aids in enhancing model performance while reducing the complexity of data preparation. Second, AutoGluon integrates and stacks multiple models automatically, enhancing predictive performance. In terms of efficiency, the algorithms in AutoGluon are optimized for limited computational resources, enabling it to produce the best models within a constrained time and computational budget. One of the critical reasons for the high performance of AutoGluon is its model integration and stacking capabilities. Integration and stacking techniques combine predictions from multiple models to improve overall predictive performance.

#### 2.2.1 Bagging in AutoGluon

**Figure 1:**
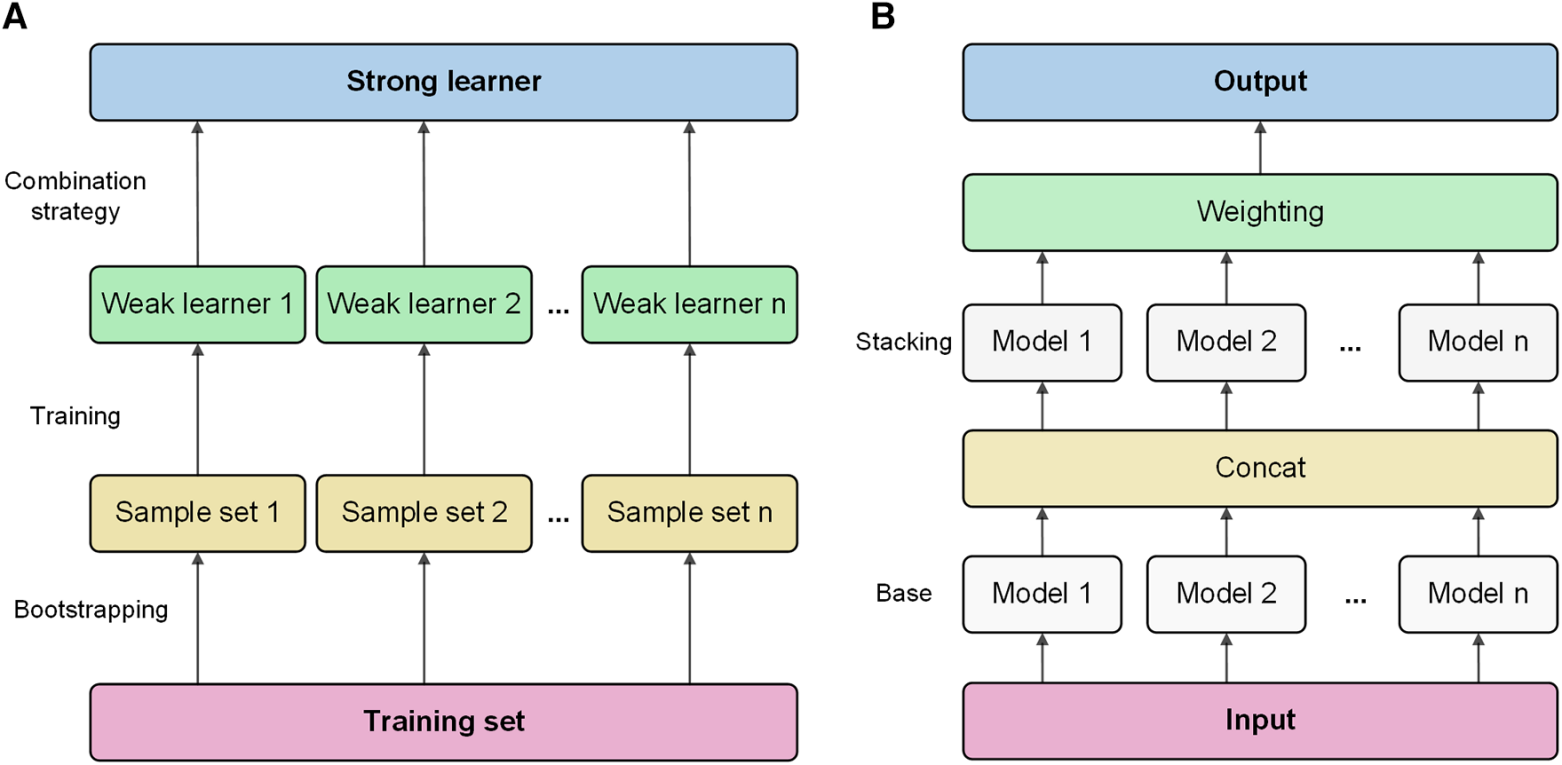
Bagging and stacking process schematic. (**A**) Training process using bagging. (**B**) Training process using stacking.

Bagging is a classic ensemble technique designed to reduce the variance of models. Bagging in AutoGluon is a fundamental ensemble method that is particularly significant for small data sets, effectively preventing model overfitting. The idea of bagging is to train independent weak learners and combine the results of each base learner to obtain one strong learner. As shown in Figure 1A, multiple data subsets of bagging are generated by repeating random sampling and replacing the original data. Bootstrapping refers to repeatedly randomly sampling with replacement from the *n* data points, sampling a total of *n* times. After *n* samplings, a new data set containing *n* samples is obtained. This data set is called a bootstrap sample. An independent model is trained on each Sample set. Choose an appropriate combination strategy to ensemble the weak learners' predictions.

#### 2.2.2 Stacking in AutoGluon

Stacking is an advanced ensemble technique wherein the output of one model is used as input features for another model. AutoGluon employs multi-level stacking, allowing each level's models to leverage the predictions of preceding models. This approach improves the model's generalization ability. The meta-learner integrates the predictions from different perspectives of different base learners, which can achieve better prediction performance than a single model. As shown in Figure 1B, stacking divides the original training data into a training set and a reserved set. Train multiple base learners on the training set. Base learners predict the hold-out set, and these predictions are spliced into new features. Optionally weight the base learner predictions. Train a meta-learner on the predicted features and real labels of the hold-out set as new samples.

### 2.3 Baseline models

The baseline models used in this study are decision tree, LightGBM, CatBoost, XGBoost, random forest, KNN, neural networks, and FastAI.

Decision tree is a classification algorithm based on a tree-like structure, which classifies data by partitioning the feature space into independent regions. Decision tree is a classification algorithm based on tree structure, which is easy to understand and explain, insensitive to outliers, but easy to overfit. LightGBM is a gradient lifting framework, which is famous for its efficient training speed and accuracy, but it may be over-fitted to small-scale data sets and needs more tuning parameters (28). CatBoost is specially designed for processing classification features, which can automatically process the coding of classification features, but the training speed is slow, and the requirements for superparameter tuning are high (29). XGBoost uses pre-sorting technology and regularization to improve the performance and stability of the model, but it needs more tuning parameters for large-scale data sets (30). Random forest is an ensemble learning algorithm that performs classification or regression by aggregating the votes of multiple decision trees. K-nearest neighbors (KNN) is an instance-based learning algorithm that performs classification or regression by finding the nearest K neighbors in the training data. KNN does not need a training process, but the computational complexity of large-scale data sets is high. Neural networks are models composed of multiple layers of neurons optimized using the backpropagation algorithm. FastAI is a deep learning library based on PyTorch, which provides an easy-to-use API and is suitable for quickly building and training deep learning models. However, it may not support some advanced functions and needs additional customization and adjustment (31).

### 2.4 SHAP: model explanation

SHAP is a method for explaining model predictions based on the Shapley value concept in game theory (32). The calculation of Shapley values utilized the formula provided in Supplementary Material Section 1. It assesses various combinations of feature values to quantify the influence of each feature on the model's predictions, providing a numerical value that represents the degree of contribution of each feature to the predicted value of a specific sample. A positive number indicates a positive impact on the result, while a negative number indicates a negative impact. SHAP can explain linear models, tree models, neural networks, and so on. It provides an intuitive graphical display way to help users better understand the prediction process of the model and reveal the influence degree of each feature in the model on the results.

SHAP offers a systematic framework for estimating feature importance and ensures the consistency and fairness of the results. This study utilizes SHAP to explain the CAD prediction model.

### 2.5 Experimental process

**Figure 2:**
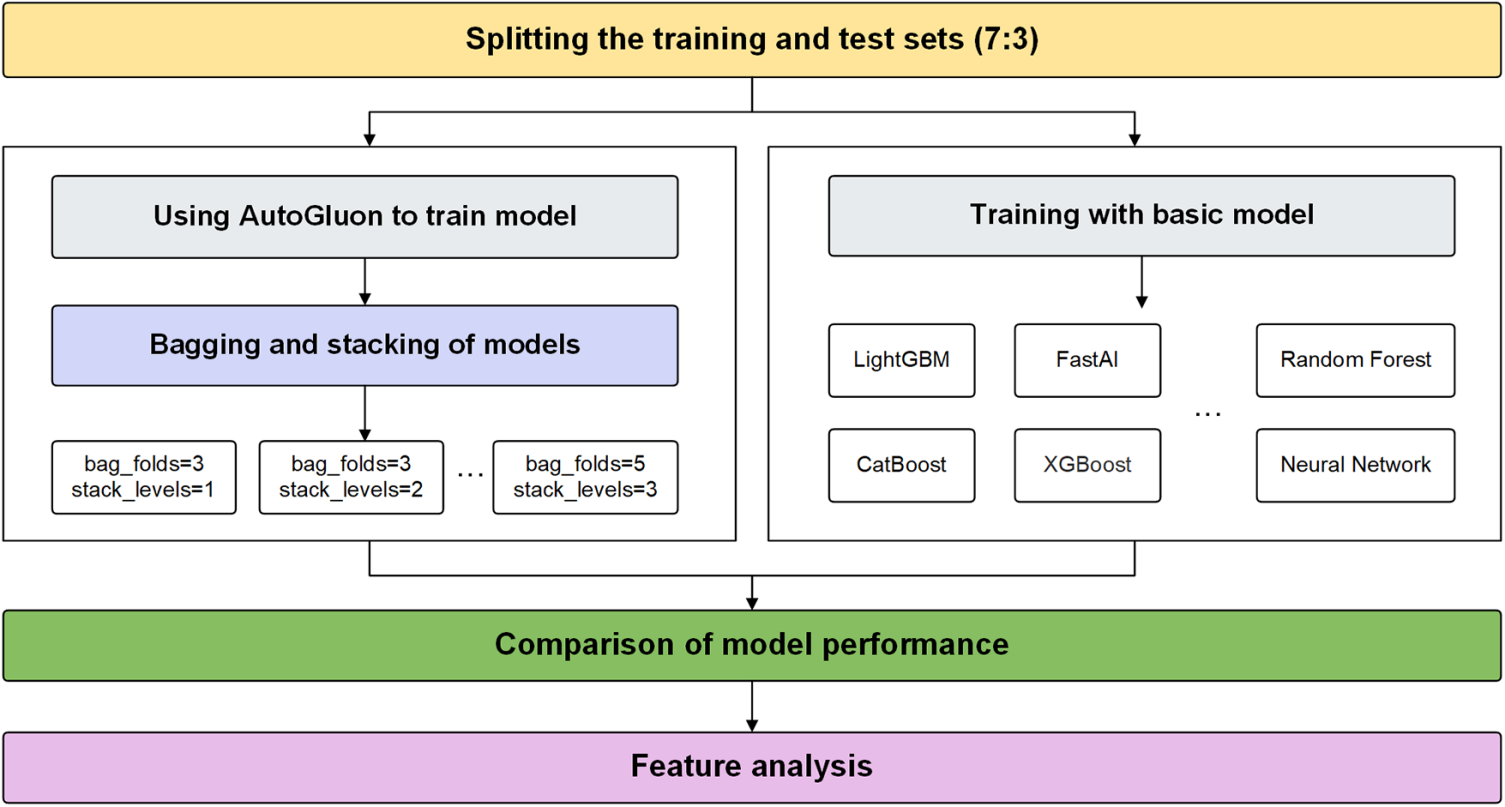
The experimental process.

This study proposes an AutoGluon-based model for predicting CAD and analyzes its explainability using SHAP. Experiments are conducted to determine the optimal AutoGluon model by tuning two key parameters, stack-level and bag-fold. Stack-level determines the depth of the layers for stacking learning, while bag-fold specifies the number of folds used in bagging. The experimental flow proposed in this study is shown in Figure 2.

The data set is divided into a 7-3 ratio, with 70% as the training set and 30% as the test set. Firstly, we build an ensemble model of CAD prediction based on AutoGluon. AutoGluon uses bagging and stacking to integrate baseline models. Then, we test the training results of each ensemble model to determine the best parameters. The key code snippets are provided in Supplementary Material Section 2. Outside of Autogluon, we train using the eight individual machine learning models. The performances of these models are compared with the AutoGluon. Finally, feature analysis is carried out based on SHAP.

## 3 Results and analysis

### 3.1 Descriptive statistical analysis

#### 3.1.1 Qualitative data analysis

**Figure 3:**
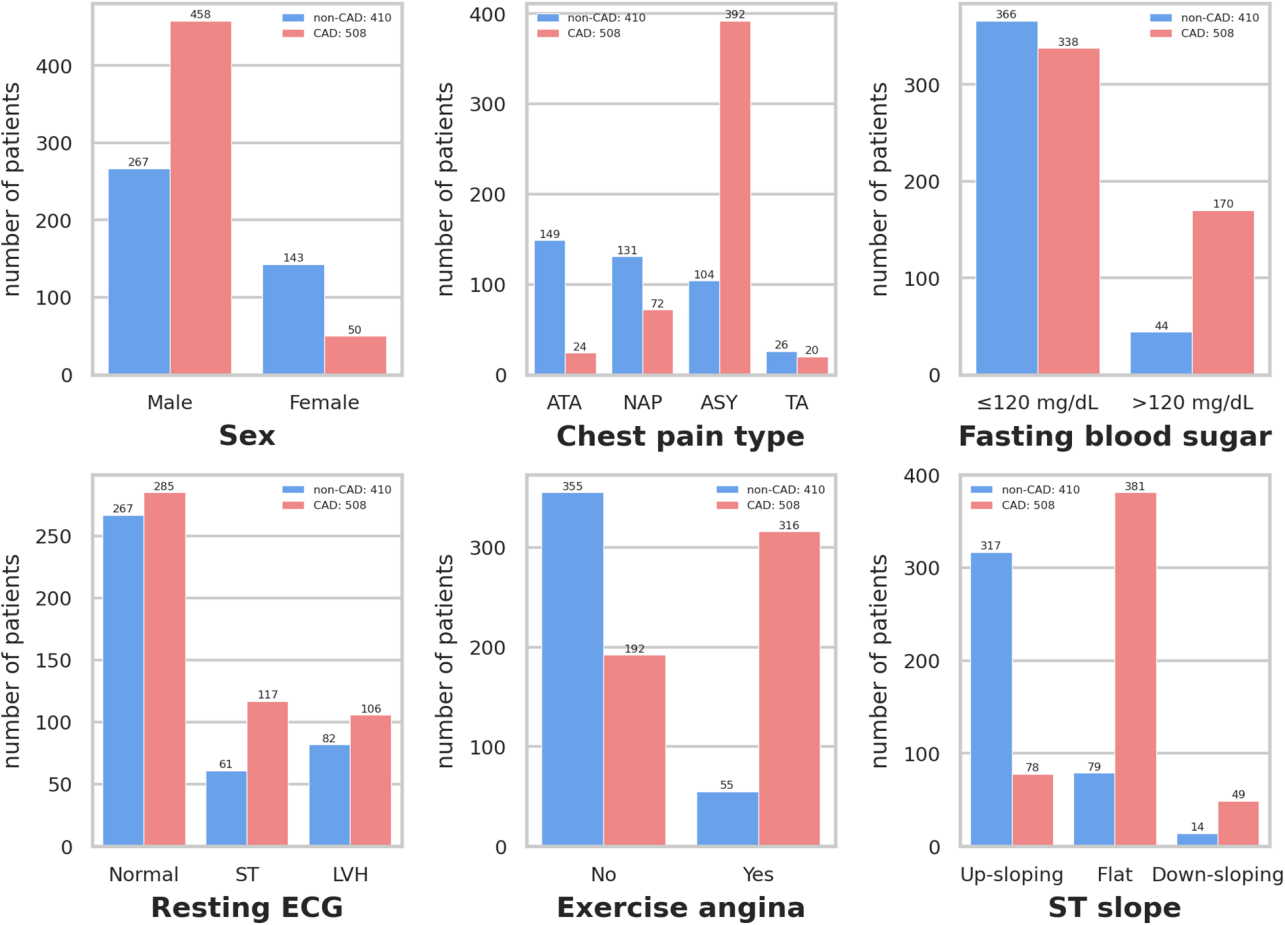
Distribution of qualitative data. Resting ECG: Resting electrocardiogram results. Exercise angina: Exercise-induced angina. ST slope: The slope of the peak exercise ST segment. ATA: Atypical angina. NAP: Non-anginal pain. ASY: Asymptomatic. TA: Typical angina. ST: Having ST-T wave abnormality (T wave inversions and/or ST elevation or depression of >0.05 mV). LVH: Showing probable or definite left ventricular hypertrophy by Estes’ criteria.

Figure 3 depicts that variables exhibiting pronounced distributional differences between CAD and non-CAD patients included ST slope, chest pain type, and exercise angina among all categorical data. In contrast, resting ECG displayed a similar distributional pattern between CAD and non-CAD patients. In non-CAD patients, there is a higher proportion of males than females. This gender disparity further increases in CAD patients. Of individuals diagnosed with CAD, 75.0% exhibit a “flat” ST slope, 77.2% have a chest pain type of “Asymptomatic,” and 62.2% of CAD patients experience “yes” for exercise angina. In contrast, the predominant ST slope among those without CAD is “up-sloping,” accounting for 77.3%. Additionally, 86.6% of non-CAD patients showed “no” for exercise angina.

#### 3.1.2 Quantitative data analysis

**Figure 4:**
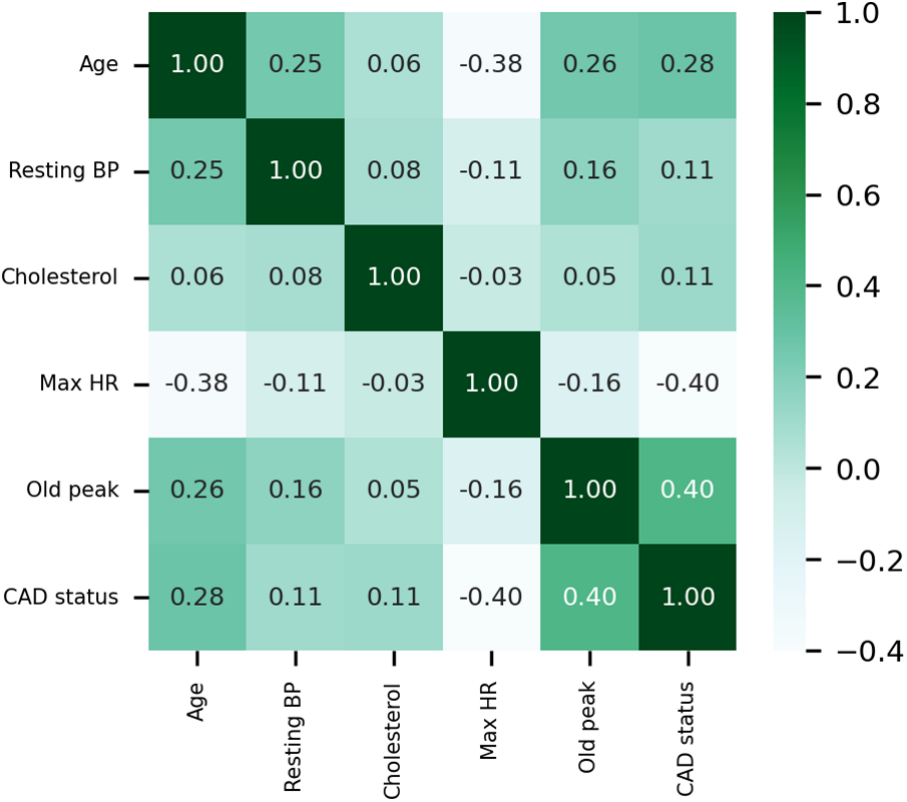
Heat map of the correlations of quantitative data. Resting BP: Resting blood pressure. Max HR: Maximum heart rate achieved.

The Pearson correlation coefficient is employed to elucidate the interrelationships between variables, and the correlation coefficient heat map among the variables is presented in Figure 4. Maximum heart rate achieved (max HR) and ST depression induced by exercise relative to rest (old peak) exhibit the strongest correlations with CAD status among all quantitative variables. CAD status demonstrates an inverse correlation with max HR, yielding a correlation coefficient of −0.4, while exhibiting a positive correlation with old peak, with a correlation coefficient 0.4. The correlation coefficient between CAD status and age is 0.28, while the correlation coefficient between CAD status and cholesterol is 0.11. Moreover, the correlation coefficient between resting blood pressure (resting BP) and CAD status is also 0.11.

**Figure 5:**
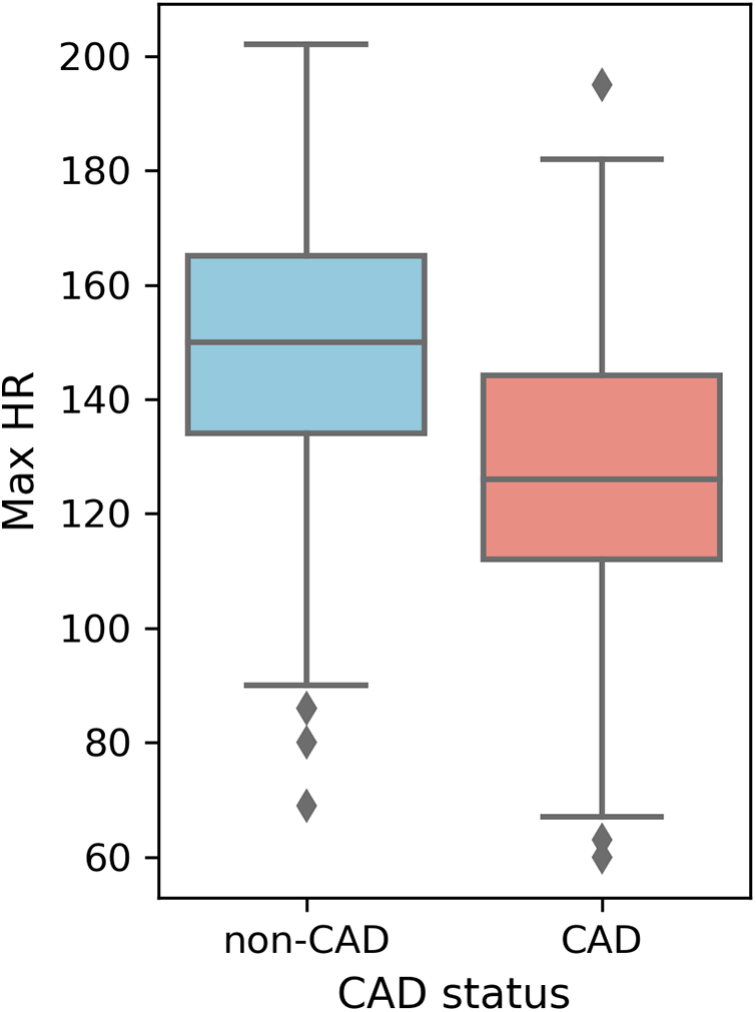
Box plot of max HR distribution. Max HR: Maximum heart rate achieved.

**Figure 6:**
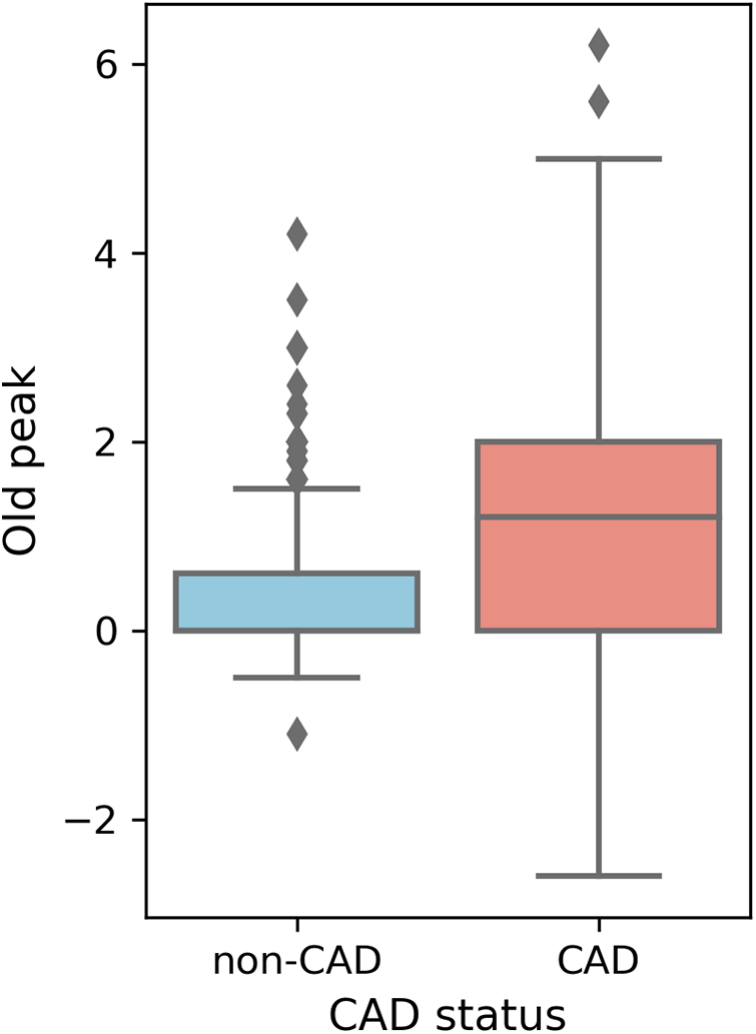
Box plot of old peak distribution. Old peak: ST depression induced by exercise relative to rest.

Higher overall max HR values are among non-CAD patients compared to those with CAD. Max HR values for non-CAD patients are primarily distributed between 140 and 160, whereas CAD patients exhibit max HR distributions predominantly between 110 and 140 (Figure 5). Additionally, distributions of old peak values are inspected. Old peak values for non-CAD patients display a concentrated range, primarily distributed between 0 and 1, while CAD patients exhibit a more comprehensive old peak range, chiefly distributed from 0 to 2 (Figure 6). The distributions of max HR and old peak exhibit pronounced differences between patients with and without CAD, implying that these features may be of substantial importance for developing a predictive model for CAD.

### 3.2 Model performance

#### 3.2.1 AutoGluon model

After detailed model training and comparison, the experiment analyzes the performance of the AutoGluon model to evaluate its effectiveness in predicting CAD.

As shown in Table 3, the experiment examines nine different parameter combinations of the AutoGluon model. Notably, when the bag-fold is set to 4 among these combinations, implying the utilization of 4-fold cross-bagging, the model exhibits the highest accuracy, reaching 0.9167. More specifically, when the parameters are set to stack-level of 1 and bag-fold of 4, the model outperforms other parameter combinations regarding key performance indicators, such as accuracy, recall, F1-score, and AUC. The precision metric is only slightly inferior to the model parameter settings of stack-level of 3 and bag-fold of 5, indicating that the AutoGluon model under this parameter combination demonstrates outstanding performance in CAD prediction.

**Table 3:** Performance of autoGluon models with nine parameter combinations.

Stack-level	Bag-fold	Accuracy	Precision	Recall	F1-score	AUC
1	3	0.8877	0.9325	0.8837	0.9074	0.9487
1	4	0.9167	0.9461	0.9186	0.9322	0.9562
1	5	0.8986	0.9444	0.8895	0.9162	0.9526
2	3	0.8986	0.9337	0.9012	0.9172	0.9475
2	4	0.9167	0.9461	0.9186	0.9322	0.9424
2	5	0.8877	0.9433	0.8721	0.9063	0.9388
3	3	0.8986	0.9337	0.9012	0.9172	0.9475
3	4	0.9167	0.9461	0.9186	0.9322	0.9424
3	5	0.8949	0.9497	0.8779	0.9124	0.9388

Accuracy: Proportion of correctly classified samples to the total samples;

Precision: Of the predicted positives, the proportion that is actually positive;

Recall: Of the actual positives, the proportion that is predicted positive;

F1-score: the scores considering both precision and recall;

AUC, area under the curve.

Overall, all nine different parameter combinations of the AutoGluon model achieve relatively high scores across the five evaluation metrics. This attests to the robust performance of the AutoGluon model and confirms its high reliability in automating the assessment of CAD prediction.

#### 3.2.2 Comparison of model prediction

**Figure 7:**
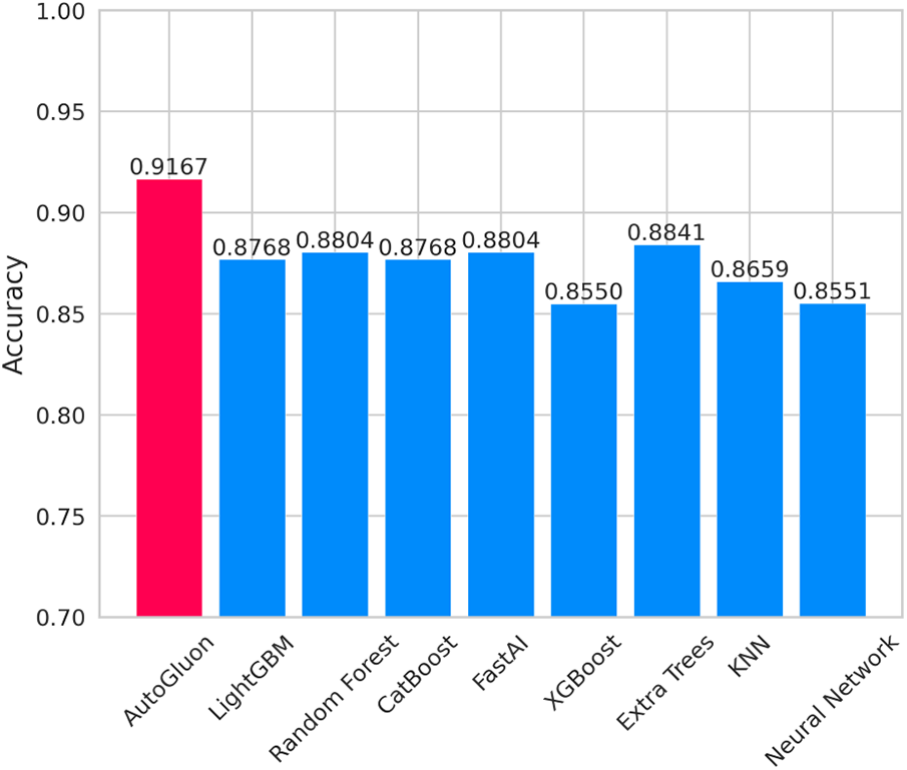
Accuracy of autoGluon and baseline models.

Figure 7 lists the prediction accuracy of each base model for CAD prediction. Based on the data set used in this study, the prediction accuracy of these eight baseline models exceeds 0.85. Among these models, LightGBM and CatBoost stand out with exemplary performance, achieving a prediction accuracy of up to 0.8768. However, despite the relatively high prediction accuracy shown by these baseline models, their accuracy rates are lower than that achieved using the multi-model ensemble method in AutoGluon, indicating that AutoGluon's multi-model ensemble approach outperforms the individual baseline models in CAD prediction.

### 3.3 Feature analysis

In complex predictive models, a deep investigation into feature importance is pivotal to understanding the behavior of the model. In this study, SHAP is utilized to explain the ensemble model built by AutoGluon with parameters bag-fold of 4 and stack-level of 1.

**Figure 8:**
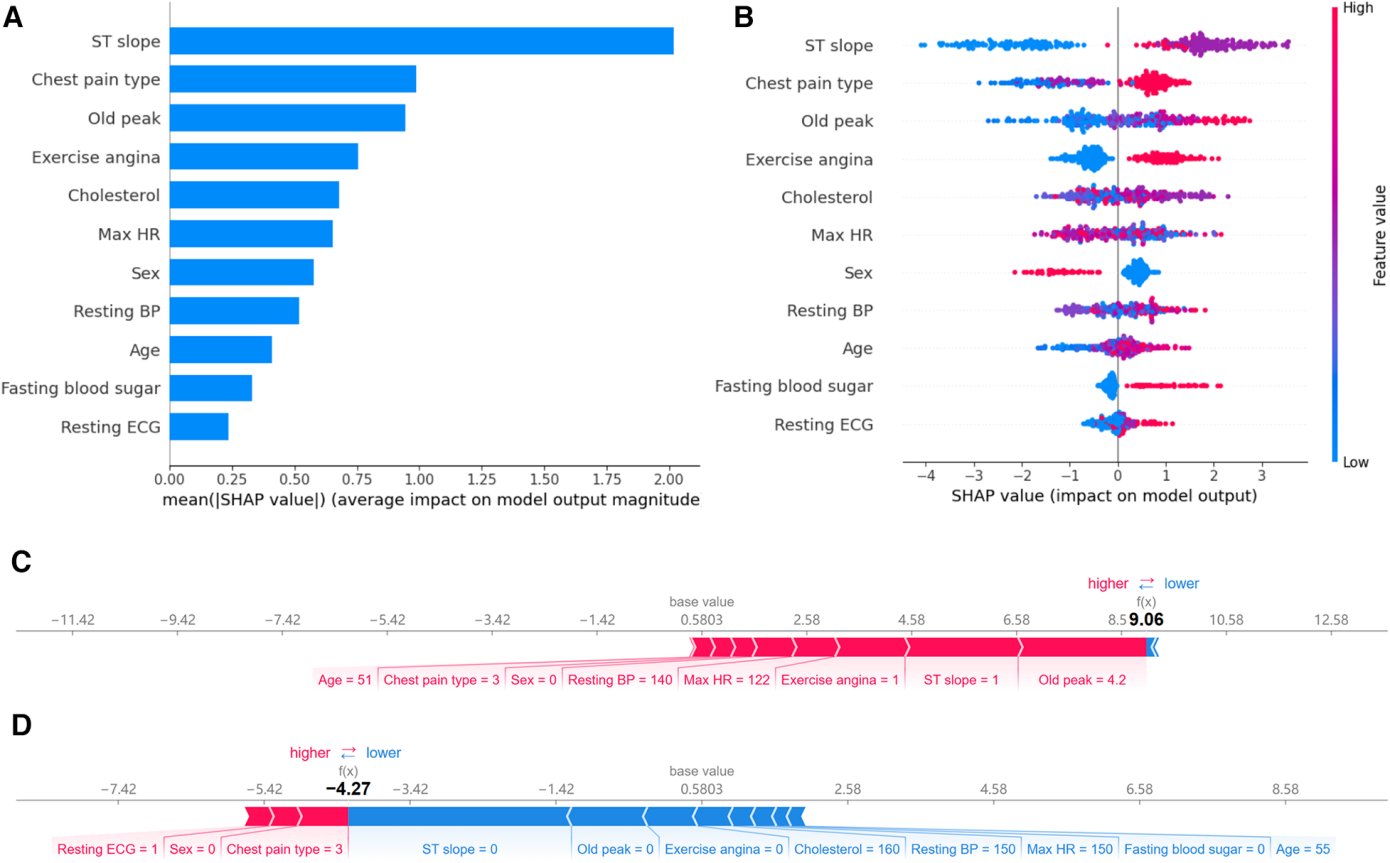
Feature importance analysis based on SHAP. (**A**) Ranking of feature importance: Ranked by the average SHAP values of 11 features. (**B**) Positive and negative impacts of features on model predictions: Features to the left of the central line negatively influence the predictions, while those to the right positively influence the predictions. (**C**) Individualized prediction interpretations for samples with CAD: The base value represents the baseline prediction made without any feature contributions. Arrows depict the impact of each feature on the prediction, with blue arrows indicating a decrease in risk and red arrows indicating an increase in risk. (**D**) Individualized prediction interpretations for samples without CAD.

Feature importance provides an effective method to understand and interpret the decisions made by a model. By quantifying the contribution of each feature to the model's prediction outcome, we can gain insights into the relative importance of the different inputs and their roles in the decision-making process. In qualitative data, ST slope, chest pain type, and exercise angina have a significant impact on the model's predictions (Figure 8A). For the quantitative data, old peak has the most significant influence on model prediction. It consistent with their correlations with CAD risk shown in the heat map.

While a straightforward ranking of feature importance offers insights into model decision-making, it still needs to elucidate the model's intricate decision patterns fully. SHAP visualization provides an additional layer of explainability. Based on feature importance, it arranges the features around a centerline. Based on each feature's SHAP value, samples are marked at their respective coordinate positions. Features on the left of the centerline have a negative SHAP value, indicating a pull towards a negative prediction. In contrast, those on the right side have a positive value, leading the model's prediction towards a positive outcome. Moreover, each sample point is color-coded: red indicates higher feature values, while blue suggests lower values. This visualization lets us perceive how varying feature values influence the model's prediction direction.

Figure 8B reveals the following features. Firstly, when the ST slope is “flat,” the model tends to predict the sample as a CAD patient. According to the distribution in Figure 2, 75.0% of CAD patients have an ST slope value of 1 (flat), while 77.3% of non-CAD patients have an ST slope value of 0 (up-sloping). It can be observed that the algorithm's predictions may be related to the differences in the distribution of ST slope values in the population. Similarly, different values of chest pain type and exercise angina also affect the model's predictions. Finally, as shown in Figure 8B, the model tends to predict male samples as CAD patients more than female samples.

The SHAP values showcase the contribution of each feature towards the final prediction, facilitating a lucid understanding and explanation of an individual patient's model prediction. Figures 8C,D illustrate two typical examples elucidating the model's individualized predictions: one sample of a patient diagnosed with CAD and another of a patient without CAD. The arrows indicate the influence of each factor on the prediction. The arrow's color determines whether the factor decreases (blue) or increases (red) the mortality risk. The cumulative influence of all factors provides the final SHAP value, correlating with the prediction score. For the model constructed in this study, the base value is 0.5803. For the first sample, the model output value is relatively high, at 9.06, indicating that the model considers the possibility of CAD in this sample relatively high. The model tends to diagnose this sample as CAD based on the values of old peak, ST slope, exercise angina, max HR, resting BP and so on. In contrast, for the second sample, the model output value is lower, at −4.27, suggesting that based on the values of ST slope, old peak, exercise angina, cholesterol, resting BP and so on, the model believes the probability of CAD in this sample is small.

## 4 Discussion

This study validates the effectiveness of AutoGluon in coronary artery disease prediction on the public data set, especially its method of integrating multiple models surpassing singular foundational models. There are inherent limitations associated with the data set used in our study, including significant differences and a lack of propensity matching, which could potentially hinder the effectiveness of our models. Moreover, there is a lack of distinction between prevalent and incident cases, as well as a deficiency in appropriate data on potential confounders, further contributing to the limitations of this data set.

In existing research, single machine learning methods are commonly utilized or further optimized, such as KNN, random forest, or Bayesian-optimized XGBoost (5, 6, 12). Ayatollahi et al. compared the predictive effects of artificial neural networks and SVM on coronary artery disease (33). The results indicated that the SVM exhibited higher accuracy and superior performance compared to the artificial neural networks model. Abdar et al. introduced a novel optimization technique, N2Genetic optimizer, for enhancing SVM. Research findings demonstrated that the proposed method for optimizing machine learning-based approaches could be successfully applied to raw data, leading to the development of predictive models for clinical and research purposes (34). Agrawal et al. developed a framework utilizing elastic net regularized Cox regression to select 51 coronary artery disease risk prediction factor subsets from 13,782 features. The model demonstrated better predictive performance in the test cohort compared to algorithms used clinically (35). Wang et al. constructed a predictive model using random forest and evaluated the model using ROC curves. This model integrated 15 indicators to assess coronary artery disease risk and exhibited robust predictive capability (36). The cloud-random forest model, which combines cloud model and random forest, is used to evaluate coronary artery disease risk. Results indicated a classification accuracy of 85% on their experimental data set, outperforming other methods in coronary artery disease risk assessment in terms of classification performance and effectiveness (37). Some studies employed ensemble learning to build models, selecting multiple machine learning algorithms as base learners, optimizing each base learner, and choosing appropriate strategies to combine predictions. Shorewala et al. utilized ensemble techniques to enhance coronary artery disease prediction accuracy. The bagging model demonstrated an average accuracy improvement of 1.96% compared to traditional models, while the boosting model achieved the highest AUC score (38). Trigka et al. experimentally evaluated various machine learning models after employing SMOTE. The results demonstrated that the stacked ensemble model post-SMOTE outperformed other models, achieving an accuracy of 90.9% (39). Kolukisa et al. proposed an ensemble feature selection method tested on publicly available data sets. Experimental results showed that the model based on ensemble feature selection achieved the best classification performance (40). Velusamy et al. introduced a novel heterogeneous ensemble method for effective diagnosis, combining three base classifiers, namely KNN, random forest, and SVM. Ensemble voting techniques based on average voting, majority voting, and weighted-average voting were utilized to combine the results of base classifiers. The results demonstrated the robustness of ensemble algorithms in both coronary artery disease patients and healthy subjects (41).

Different machine learning models may exhibit varying performance on specific data sets. Building machine learning models requires researchers to manually select algorithms, tune hyperparameters, perform feature selection, and preprocess data, among other steps. This study leverages AutoML technology to automate the construction of coronary artery disease prediction models. Results demonstrate that AutoGluon-based models consistently outperform those built using individual machine learning methods on CAD prediction tasks.

In the medical field, the application of AutoML is attracting increasing attention and showing tremendous potential in various aspects. The main advantage of AutoML lies in its ability to simplify the construction and optimization process of machine learning models, thereby accelerating the speed of medical data analysis and model development, reducing technical barriers, and improving model performance. AutoML can be applied to disease diagnosis and personalized prediction. For example, it can be used to enhance the detection of sinus diseases and predict acute kidney injury in acute pancreatitis (42, 43). The application of AutoML in the medical field holds vast prospects and is expected to have a profound impact on medical research and clinical practice.

While AutoML demonstrates excellent performance in cardiovascular disease prediction, model explainability is equally essential in the medical domain. Doctors, when making decisions, not only rely on the predictive outcomes of the model but also need to understand why the model predicts as it does to integrate it with other clinical information. The role of SHAP is to explain the model's prediction results. It achieves this by providing a contribution value for each feature to explain the prediction of each sample, thereby enhancing the explainability of the model. In this study, SHAP was used to identify which features played an important role in predicting the model's results, aiding in understanding the model's decision-making process. According to the feature importance ranking plot, the features used by our model to predict coronary artery disease are ranked as follows: ST slope, chest pain type, old peak, exercise angina, cholesterol, max HR, sex, resting BP, age, fasting blood sugar, and resting ECG. According to the SHAP values, individuals with a flat or down-sloping ST slope have a higher risk of CAD, while those with an up-sloping ST slope have a lower risk. The SHAP value of fasting blood sugar indicates that individuals with high blood sugar levels are more likely to have CAD. This information can offer doctors additional clues in clinical practice, aiding in more precise diagnosis and treatment.

Regarding the limitations of this study, it is important to note that the data set utilized originates from open-source websites and is limited in scope. While AutoGluon demonstrates superior performance compared to other foundational models, it is essential to understand that this does not necessarily mean it is the optimal choice for all medical tasks. Furthermore, in terms of feature importance, although SHAP offers a method, further research is needed to explore its generalizability across different data sets and models.

In light of the limitations mentioned above, future research should consider the use of larger and more diverse data sets or explore the application of techniques such as few-shot learning, transfer learning, and active learning to address the challenge posed by the small size of the data set, thereby enhancing the model's generalization capability (44–46). Additionally, it is crucial to explore and compare other AutoML frameworks and algorithms, such as H2O AutoML and TPOT, and conduct in-depth comparisons with AutoGluon to determine optimal strategies for specific applications (47, 48). The explainability of machine learning models remains a pivotal issue, necessitating more in-depth validation across different medical domains to ensure its consistency with clinical practice (49–68).

The application of AutoML in cardiovascular medicine not only streamlines the model construction process but also paves the way for using artificial intelligence technologies in areas such as coronary artery hemodynamics and heart treatment. This ongoing exploration and application of AutoML in these domains promotes innovation, contributing to the refinement and personalization of heart treatment strategies. This enables the implementation of more effective and target intervention measures, promoting the ongoing advancement of precision medicine in the field.

## 5 Conclusion

This study proposes an explainable coronary artery disease prediction model based on the AutoML framework AutoGluon, applied in cardiovascular medicine. The model achieves optimal performance through comparative validation when adopting 4-fold cross-bagging, with an accuracy of 0.9167. Furthermore, single foundational models, such as LightGBM and CatBoost, do not surpass the predictive accuracy of the multi-model ensemble approach realized through AutoGluon. Lastly, to further comprehend the model's decision-making process, we interpret the ensemble model constructed by AutoGluon using SHAP. This study confirms the effectiveness of AutoML in disease prediction in cardiovascular medicine.

## Data Availability

The original contributions presented in the study are included in the article/**Supplementary Material**, further inquiries can be directed to the corresponding authors.

## References

[1] Tsao CW Aday AW Almarzooq ZI Anderson CAM Arora P Avery CL Heart disease and stroke statistics-2023 update: a report from the American Heart Association. Circulation. (2023) 147(8):e93–e621. 10.1161/CIR.0000000000001123 36695182 PMC12135016

[2] The Writing Committee of the Report on Cardiovascular Health and Diseases in China, Hu SS. Report on cardiovascular health and diseases in China 2021: an updated summary. J Geriatr Cardiol. (2023) 20(6):399–430. 10.26599/1671-5411.2023.06.001 37416519 PMC10320777

[3] Jordan MI Mitchell TM. Machine learning: trends, perspectives, and prospects. Science. (2015) 349(6245):255–60. 10.1126/science.aaa8415 26185243

[4] Deo RC. Machine learning in medicine. Circulation. (2015) 132(20):1920–30. 10.1161/CIRCULATIONAHA.115.001593 26572668 PMC5831252

[5] Shah D Patel S Bharti SK. Heart disease prediction using machine learning techniques. SN Computer Science. (2020) 1:1–6. 10.1007/s42979-020-00365-y

[6] Sharma V Yadav S Gupta M. Heart disease prediction using machine learning techniques. 2020 2nd International Conference on Advances in Computing, Communication Control and Networking (ICACCCN); *2020 Dec 18–19; Greater Noida, India*. New York, NY: IEEE (2020).

[7] Dwivedi AK. Performance evaluation of different machine learning techniques for prediction of heart disease. Neural Comput Appl. (2018) 29:685–93. 10.1007/s00521-016-2604-1

[8] Nandy S Adhikari M Balasubramanian V Menon VG Li X Zakarya M. An intelligent heart disease prediction system based on swarm-artificial neural network. Neural Comput Appl. (2023) 35(20):14723–37. 10.1007/s00521-021-06124-1

[9] Khourdifi Y Baha M. Heart disease prediction and classification using machine learning algorithms optimized by particle swarm optimization and ant colony optimization. Int J Intell Engineer Syst. (2019) 12(1):242–52. 10.22266/ijies2019.0228.24

[10] Asadi S Roshan S Kattan MW. Random forest swarm optimization-based for heart diseases diagnosis. J Biomed Inform. (2021) 115:103690. 10.1016/j.jbi.2021.103690 33540075

[11] Cherian RP Thomas N Venkitachalam S. Weight optimized neural network for heart disease prediction using hybrid lion plus particle swarm algorithm. J Biomed Inform. (2020) 110:103543. 10.1016/j.jbi.2020.103543 32858167

[12] Budholiya K Shrivastava SK Sharma V. An optimized Xgboost based diagnostic system for effective prediction of heart disease. J King Saud Univ Comp Inform Sci. (2022) 34(7):4514–23. 10.1016/j.jksuci.2020.10.013

[13] Latha CBC Jeeva SC. Improving the accuracy of prediction of heart disease risk based on ensemble classification techniques. Inform Med Unlocked. (2019) 16:100203. 10.1016/j.imu.2019.100203

[14] Lakshmanarao A Srisaila A Kiran TSR. Heart disease prediction using feature selection and ensemble learning techniques. 2021 Third International Conference on Intelligent Communication Technologies and Virtual Mobile Networks (ICICV); *2021 Feb 04–06; Tirunelveli, India*. New York, NY: IEEE (2021).

[15] Raza K. Improving the prediction accuracy of heart disease with ensemble learning and majority voting rule. In: In U-Healthcare Monitoring Systems. Amsterdam: Elsevier (2019). p. 179–96.

[16] Mohan S Thirumalai C Srivastava G. Effective heart disease prediction using hybrid machine learning techniques. IEEE access. (2019) 7:81542–54. 10.1109/ACCESS.2019.2923707

[17] Truong A Walters A Goodsitt J Hines K Bruss CB Farivar R, editors. Towards automated machine learning: evaluation and comparison of automl approaches and tools. 2019 IEEE 31st International Conference on Tools with Artificial Intelligence (ICTAI); 2019: IEEE.

[18] He X Zhao K Chu X. AutoML: a survey of the state-of-the-art. Knowl Based Syst. (2021) 212:106622. 10.1016/j.knosys.2020.106622

[19] Ikemura K Bellin E Yagi Y Billett H Saada M Simone K Using automated machine learning to predict the mortality of patients with COVID-19: prediction model development study. J Med Internet Res. (2021) 23(2):e23458. 10.2196/23458 33539308 PMC7919846

[20] Yu C Li Y Yin M Gao J Xi L Lin J Automated machine learning in predicting 30-day mortality in patients with non-cholestatic cirrhosis. J Pers Med. (2022) 12(11):1930. 10.3390/jpm12111930 36422105 PMC9693570

[21] Yin M Zhang R Zhou Z Liu L Gao J Xu W Automated machine learning for the early prediction of the severity of acute pancreatitis in hospitals. Front Cell Infect Microbiol. (2022) 12:886935. 10.3389/fcimb.2022.886935 35755847 PMC9226483

[22] Shi Y Lin J Zhu J Gao J Liu L Yin M Predicting the recurrence of common bile duct stones after ercp treatment with automated machine learning algorithms. Dig Dis Sci. (2023) 68(7):1–12. 10.1007/s10620-023-07949-7 37160541

[23] SenthilKumar G Madhusudhana S Flitcroft M Sheriff S Thalji S Merrill J Automated machine learning (automl) can predict 90-day mortality after gastrectomy for cancer. Sci Rep. (2023) 13(1):11051. 10.1038/s41598-023-37396-3 37422500 PMC10329647

[24] Markus AF Kors JA Rijnbeek PR. The role of explainability in creating trustworthy artificial intelligence for health care: a comprehensive survey of the terminology, design choices, and evaluation strategies. J Biomed Inform. (2021) 113:103655. 10.1016/j.jbi.2020.103655 33309898

[25] Tjoa E Guan C. A survey on explainable artificial intelligence (Xai): toward medical Xai. IEEE Trans Neural Netw Learn Syst. (2020) 32(11):4793–813. 10.1109/TNNLS.2020.3027314 33079674

[26] Siddhartha M. Heart disease data set (comprehensive). IEEE Dataport. (2020). 10.21227/dz4t-cm36

[27] Erickson N Mueller J Shirkov A Zhang H Larroy P Li M Autogluon-Tabular: Robust and Accurate Automl for Structured Data. *arXiv* [preprint]. *arXiv:200306505* (2020). 10.48550/arXiv.2003.06505

[28] Ke G Meng Q Finley T Wang T Chen W Ma W Lightgbm: a highly efficient gradient boosting decision tree. Advances in Neural Information Processing Systems; 2017 Dec 04–09; Long Beach, CA, USA. San Diego, CA: NIPS (2017). p. 30.

[29] Prokhorenkova L Gusev G Vorobev A Dorogush AV Gulin A. Catboost: unbiased boosting with categorical features. Adv Neural Inf Process Syst. (2018) 31:6639–49.

[30] Chen T Guestrin C, editors. Xgboost: a scalable tree boosting system. Proceedings of the 22nd acm Sigkdd International Conference on Knowledge Discovery and Data Mining; 2016.

[31] Howard J Gugger S. Fastai: a layered api for deep learning. Information. (2020) 11(2):108. 10.3390/info11020108

[32] Lundberg SM Lee S-I. A unified approach to interpreting model predictions. Advances in Neural Information Processing Systems; 2017 Dec 04–09; Long Beach, CA, USA. San Diego, CA: NIPS (2017). p. 30.

[33] Ayatollahi H Gholamhosseini L Salehi M. Predicting coronary artery disease: a comparison between two data mining algorithms. BMC Public Health. (2019) 19:1–9. 10.1186/s12889-019-6721-5 31035958 PMC6489351

[34] Abdar M Książek W Acharya UR Tan R-S Makarenkov V Pławiak P. A new machine learning technique for an accurate diagnosis of coronary artery disease. Comput Methods Programs Biomed. (2019) 179:104992. 10.1016/j.cmpb.2019.104992 31443858

[35] Agrawal S Klarqvist MD Emdin C Patel AP Paranjpe MD Ellinor PT Selection of 51 predictors from 13,782 candidate multimodal features using machine learning improves coronary artery disease prediction. Patterns. (2021) 2(12):1–12. 10.1016/j.patter.2021.100364 PMC8672148 34950898

[36] Wang C Zhao Y Jin B Gan X Liang B Xiang Y Development and validation of a predictive model for coronary artery disease using machine learning. Front Cardiovasc Med. (2021) 8:614204. 10.3389/fcvm.2021.614204 33634169 PMC7902072

[37] Wang J Rao C Goh M Xiao X. Risk assessment of coronary heart disease based on cloud-random forest. Artif Intell Rev. (2023) 56(1):203–32. 10.1007/s10462-022-10170-z

[38] Shorewala V. Early detection of coronary heart disease using ensemble techniques. Inform Med Unlocked. (2021) 26:100655. 10.1016/j.imu.2021.100655

[39] Trigka M Dritsas E. Long-term coronary artery disease risk prediction with machine learning models. Sensors. (2023) 23(3):1193. 10.3390/s23031193 36772237 PMC9920214

[40] Kolukisa B Bakir-Gungor B. Ensemble feature selection and classification methods for machine learning-based coronary artery disease diagnosis. Comp Stand Inter. (2023) 84:33–40. 10.1016/j.csi.2022.103706

[41] Velusamy D Ramasamy K. Ensemble of heterogeneous classifiers for diagnosis and prediction of coronary artery disease with reduced feature subset. Comput Methods Programs Biomed. (2021) 198:105770. 10.1016/j.cmpb.2020.105770 33027698

[42] Cheong RCT Jawad S Adams A Campion T Lim ZH Papachristou N Enhancing paranasal Sinus disease detection with automl: efficient ai development and evaluation via magnetic resonance imaging. Eur Arch Oto-Rhino-Laryngol. (2024) 281:2153–8. 10.1007/s00405-023-08424-9 PMC10942883 38197934

[43] Zhang R Yin M Jiang A Zhang S Xu X Liu L. Automated machine learning for early prediction of acute kidney injury in acute pancreatitis. BMC Med Inform Decis Mak. (2024) 24(1):16. 10.1186/s12911-024-02414-5 38212745 PMC10785491

[44] Chen W-Y Liu Y-C Kira Z Wang Y-CF Huang J-B. A Closer Look at Few-Shot Classification. *arXiv* [Preprint]. *arXiv:190404232* (2019). 10.48550/arXiv.1904.04232

[45] Weiss K Khoshgoftaar TM Wang D. A survey of transfer learning. J Big Data. (2016) 3(1):1–40. 10.1186/s40537-016-0043-6

[46] Ren P Xiao Y Chang X Huang P-Y Li Z Gupta BB A survey of deep active learning. ACM Computing Surveys (CSUR). (2021) 54(9):1–40. 10.1145/3472291

[47] LeDell E Poirier S, editors. H2so automl: scalable automatic machine learning. Proceedings of the AutoML Workshop at ICML; 2020: ICML.

[48] Olson RS Moore JH, editors. Tpot: a tree-based pipeline optimization tool for automating machine learning. Workshop on Automatic Machine Learning; 2016: PMLR.

[49] Liu Z Ma C Gu J Yu M. Potential biomarkers of acute myocardial infarction based on weighted gene co-expression network analysis. Biomed Eng Online. (2019) 18:1–12. 30683112 10.1186/s12938-019-0625-6 PMC6347746

[50] Ren G Liu Z. NetCAD: a network analysis tool for coronary artery diseaseassociated PPI network. Bioinform. (2013) 29(2):279–80. 10.1093/bioinformatics/bts666 23162052

[51] Gong X Liu L Fong S Xu Q Wen T Liu Z. Comparative research of swarm intelligence clustering algorithms for analyzing medical data. IEEE Access. (2019) 7:137560–9.

[52] Liu Z Chu G. Chronobiology in mammalian health. Mol Biol Rep. (2013) 40:2491–501. 23224435 10.1007/s11033-012-2330-4

[53] Cheng J Zeng X Ren G Liu Z. CGAP: a new comprehensive platform for the comparative analysis of chloroplast genomes. BMC Bioinformatics. (2013) 14:1–8. 23496817 10.1186/1471-2105-14-95 PMC3636126

[54] Cheng J Cao F Liu Z. AGP: a multimethods web server for alignment-free genome phylogeny. Mol Biol Evol. (2013) 30(5):1032–7. 23389766 10.1093/molbev/mst021 PMC7574599

[55] Yuan Z Zeng X Yang D Wang W Liu Z. Effects of common polymorphism rs11614913 in Hsa-miR-196a2 on lung cancer risk. PLoS One. (2013) 8(4):e61047. 23593385 10.1371/journal.pone.0061047 PMC3625214

[56] Hu R Zeng X Gao W Wang Q Liu Z. HRAS: a webserver for early warning of human health risk brought by aflatoxin. Mol Biol Rep. (2013) 40 :1181–7. 23076528 10.1007/s11033-012-2160-4

[57] Yang D Xie P Liu Z. Ischemia/reperfusion-induced MKP-3 impairs endothelial NO formation via inactivation of ERK1/2 pathway. *PLoS One.* (2012) 7(7):e42076. 22848708 10.1371/journal.pone.0042076 PMC3407110

[58] Liu Z Zeng X Yang D Ren G Chu G Yuan Z Identification of medicinal vines by ITS2 using complementary discrimination methods. J Ethnopharmacol. (2012) 141(1):242–9. 22353709 10.1016/j.jep.2012.01.057

[59] Liu Z Zeng X Yang D Chu G Yuan Z Chen S. Applying DNA barcodes for identification of plant species in the family Araliaceae. Gene. (2012) 499(1):76–80. 22406497 10.1016/j.gene.2012.02.016

[60] Zeng X Yuan Z Tong X Li Q Gao W Qin M Phylogenetic study of Oryzoideae species and related taxa of the Poaceae based on atpB-rbcL and ndhF DNA sequences. Mol Biol Rep. (2012) 39:5737–44. 22189545 10.1007/s11033-011-1383-0

[61] Yuan Z Chu G Dan Y Li J Zhang L Gao X BRCA1: a new candidate gene for bovine mastitis and its association analysis between single nucleotide polymorphisms and milk somatic cell score. Mol Biol Rep. (2012) 39:6625–31. 22327776 10.1007/s11033-012-1467-5

[62] Liu Z Sun X. Informational structure of agrobacterium tumefaciens C58 genome. International Conference on Life System Modeling and Simulation. September, Berlin, Heidelberg: Springer Berlin Heidelberg (2007). p. 153–61.

[63] Liu Z Sun X. Coronavirus phylogeny based on base-base correlation. IJBRA. (2008) 4(2):211–20. 18490264 10.1504/IJBRA.2008.018347

[64] Liu Z Liu H Li J Sun X Jiao D. Base-Base Correlation a novel sequence feature and its applications. 1st International Conference on Bioinformatics and Biomedical Engineering, IEEE, (2007). p. 370–3.

[65] Liu Z Jiao D Sun X. Classifying genomic sequences by sequence feature analysis. GPB. (2005) 3(4):201–5. 16689686 10.1016/S1672-0229(05)03027-5 PMC5172532

[66] Liu Z Meng J Sun X. A novel feature-based method for whole genome phylogenetic analysis without alignment: application to HEV genotyping and subtyping. Biochem Biophys Res Commun. (2008) 368(2):223–30. 18230342 10.1016/j.bbrc.2008.01.070

[67] Liu Z Chen S. ER regulates an evolutionarily conser ved apoptosis pathway. Biochem Biophys Res Commun. (2010) 400(1):34–8. 20691160 10.1016/j.bbrc.2010.07.132

[68] Liu Z Zhao X. PiRNAs as emerging biomarkers and physiological regulatory molecules in cardiovascular disease. Biochem Biophys Res Commun. (2024) 149906. 38640879 10.1016/j.bbrc.2024.149906

