# Supplementary material for: Explainable coronary artery disease prediction model based on AutoGluon from AutoML framework
Source: Front Cardiovasc Med. 2024 Jul 1;11:1360548. doi: 10.3389/fcvm.2024.1360548 (PMC11246996; doi:10.3389/fcvm.2024.1360548)
Supplement: Supplementary file 1 [file Datasheet1.pdf]

Supplementary Material

1 The formula for calculating Shapley value

$$\varphi_i = \sum_{S \subseteq N \setminus \{i\}} \frac{|S|!\,(|N| - |S| - 1)!}{|N|!} [\, f(S \cup \{i\}) - f(S)]$$

φ_i denotes the Shapley value for the i-th feature, N represents the set of all features, S denotes the combination of features excluding the i-th feature, $f(S)$ represents the output of the machine learning model for the input feature set S, $|N|$ indicates the number of elements in set N, $|S|$ denotes the number of elements in set S, and $f(S \cup \{i\}) - f(S)$ represents the cumulative contribution of feature i.

Similarly, in the context of machine learning, SHAP assigns a value to each feature. This value explicitly indicates the degree of influence a feature has on the model's prediction. SHAP achieves explainability by constructing an explanatory model, $g(x)$, as a substitute for a black-box model:

$$g(x) = \varphi_0 + \sum_{i=1}^{q} \varphi_i$$

q denotes the number of features, φ_0 represents the predicted mean for a set of samples, φ_i denotes the Shapley value for feature i. SHAP can intuitively identify features that play a critical role in model decisions. This could help researchers better understand the working mechanism of the model and enhance the transparency of the model, thereby increasing the credibility of the model.

2 The key code of establishing prediction model with AutoGluon

```
import pandas as pd

from autogluon.tabular import TabularDataset, TabularPredictor



label = "Target"

stacking_params = [1, 2, 3]

bagging_params = [3, 4, 5]

results = []
```

```
for stack_levels in stacking_params:

    for bag_folds in bagging_params:
        print(f"Training with stack_levels={stack_levels} and bag_folds={bag_folds}")
        predictor = TabularPredictor(label=label).fit(
            train_data,
            num_bag_folds=bag_folds,
            num_stack_levels=stack_levels,
            hyperparameters='default'
        )
        performance = predictor.evaluate(test_data)
        performance['stack_levels'] = stack_levels
        performance['bag_folds'] = bag_folds
        results.append(performance)

for performance in results:
    print(f"Stack levels: {performance['stack_levels']}, Bag folds: {performance['bag_folds']}")
    print(f"Accuracy: {performance['accuracy']}")
    print(f"Precision: {performance.get('precision', 'N/A')}")
    print(f"Recall: {performance.get('recall', 'N/A')}")
    print(f"F1: {performance.get('f1', 'N/A')}")
```
